# The Tail of the Beast

**Published:** 1882-07

**Authors:** 


### THE TAIL OF THE BEAST.

Almost every dentist who has been in the profession ten years or more, is more or less familiar with litigation and trouble to the profession, growing out of the alleged patent upon the use of vulcanite rubber for dental purposes. While the patents have expired, and the obligation arising from the alleged violation of said patent has become a thing of the past, yet a litigation, asan entailment, has existed in the State of Michigan up to the present. Some ten or twelve years ago, a few dentists in that State, formed a protective association, the object of which was to enable its members the better to resist the claims and demands of the Dental Vulcanite Co. The members of this association were comparatively few in number.

They employed an attorney who lived in Jackson, to look after their interests in this matter; to appear in the courts and make in their behalf such resistance to the demands of the Rubber Co. as was practicable. The efforts of this attorney accomplished very little in the way of protection, as I think every suit brought was decided adversely to the dentists. His efforts seemed to be largely employed in securing compensation for his service. How much he obtained is not known.

Some four years or more after the organization was formed, and after it was fully demonstrated that nothing could be done upon the plan they had been pursuing, and long after all resistance had been abandoned, the said attorney brought suit in the Circuit Court at Jackson against several dentists at Detroit and obtained a judgment against them for $15,000. This was done without any process being served upon them, or any one of them. The first they knew of the matter was by the appearance of the Sheriff of their county with an execution.

The judgment was attained for the most part against those who were not members of the Protective Union and were in no sense liable. At this point an attorney was sent to Jackson, and, upon a presentation of the facts, the judgments were set aside. Then the aforesaid attorney of the "Union" went to Detroit, and began proceedings there, and, by some means, obtained a judgment there against the same parties (one of the parties says it was a snap judgment). This was also upon a fair presentation easily set aside. After this the aforesaid plaintiff began another suit, which he wished to have delayed, but the defendants insisted that the trial should proceed. This time they were on hand and ready for the fray, and so the fight proceeded for three or four days. When the plaintiff was nonsuited, and chargeable with the costs.

This has been an exceedingly annoying procedure to the dentists of Detroit as it has caused them much loss of time, and considerable expenditure of money in the way of attorney's fees, and it was especially annoying as it detained most of the dentists of Detroit from the meetings of the State Society. Whether this is the close of the matter time only will decide.

